# Extending mechanochemical porphyrin synthesis to bulkier aromatics: tetramesitylporphyrin

**DOI:** 10.3762/bjoc.15.111

**Published:** 2019-05-22

**Authors:** Qiwen Su, Tamara D Hamilton

**Affiliations:** 1 Department of Physical Sciences, Barry University, 11300 NE 2nd Ave., Miami Shores, FL 33161, USA; 2 Department of Chemistry, Boston College, 2609 Beacon Street, Chestnut Hill, MA 02467, USA

**Keywords:** condensation, grinding, mechanochemistry, milling, porphyrin, sterically-hindered

## Abstract

Aldehydes with bulky substituents in the *ortho*-positions have been historically difficult in porphyrin synthesis, presumably owing to steric hindrance around the reactive site. We have used mechanochemistry for the simple, room-temperature synthesis of tetra-*meso*-substituted porphyrins. In the present study, mesitaldehyde undergoes acid-catalyzed mechanochemical condensation with pyrrole to give *meso*-tetrakis[2,4,6-(trimethyl)phenyl]porphyrin (TMP) after oxidation in solution. Yields are similar to those obtained using high-temperature porphyrin synthesis, although they remain significantly lower than some optimized room-temperature, solution-based methods. Yields of the mechanochemical synthesis were found to increase slightly upon longer exposure to an organic oxidizing agent in solution. This indicates that the mechanochemical condensation step may be more successful than initially realized. This work shows that mechanochemistry is a successful, simple, room-temperature method for producing tetra-*meso*-substituted porphyrins with bulky substituents.

## Introduction

**Figure 1:**
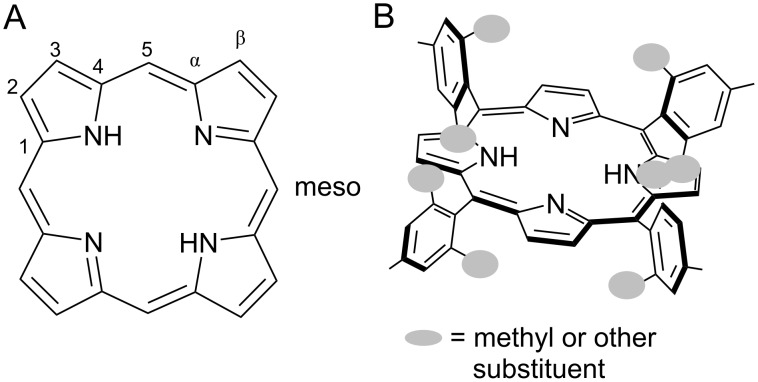
A) Porphyrin structure and labelling system. B) Substituents in the *ortho*-position of the group attached to the methane bridge create steric hindrance around the *meso*-position, above and below the plane of the porphyrin ring.

**Scheme 1:**
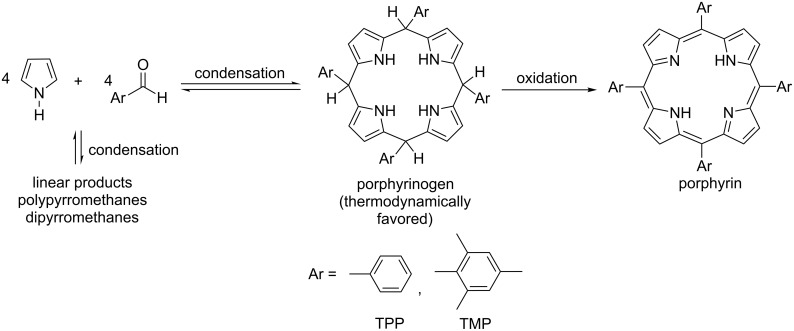
Steps leading to the formation of a porphyrin.

Porphyrins and related macrocycles such as chlorins, corroles, and bacteriochlorins carry out important functions in nature including light harvesting (i.e., chlorophyll), oxygen transport (i.e., heme), biocatalysis, and electron transfer. The ability to synthesize porphyrins bearing a variety of chemical and steric functionalities on the periphery is important in fields as diverse as catalysis [1], photovoltaics [2], photodynamic therapy [3–4], environmental remediation [5–6], biomimetic modelling [7] and metal-organic self-assembly [8]. Porphyrins may be substituted in the *meso* or β-positions (Figure 1A). Tetra-*meso*-substituted porphyrins are usually synthesized from simple starting materials, namely: pyrrole and an aldehyde (Scheme 1). Porphyrin synthesis is interesting to study as a mechanochemical reaction because it involves the combination of reactive molecules under appropriate conditions to give a very stable, aromatic product. Furthermore, depending on the nature of the substituents, the porphyrin product can be labor-intensive to produce and purify in good yield and large quantities using other methods. The process involves multiple condensations, together with oxidation that may happen in one pot or as separate steps. Other no-solvent-added methods of porphyrin synthesis have been reported [9–10], while optimization of solvent, dilution and catalysis conditions using solvent-based approaches have been investigated for the synthesis of many porphyrins [11–14].

Tetraphenylporphyrin (TPP) synthesis, as first reported in 1935 by Rothemund [15–17], involved placing reactants in a sealed tube at high temperatures (150 °C or higher) for 24 to 48 hours in the absence of air or any oxidant. Not every substituted benzaldehyde could survive this process and yield the porphyrin. Adler and Longo later introduced refluxing propionic acid (141 °C) open to air as a reaction medium [18]. This widened the variety of substituted aldehydes successfully converted to porphyrins, but employment of a caustic solvent at high temperature is a drawback. These conditions also proved inappropriate for several substituted benzaldehydes, and the best yields have been in the order of 20% with many porphyrins needing extensive work-up to isolate from tar-like byproducts. Working from the premise that the cyclized condensation product (called a porphyrinogen) should be thermodynamically favored over linear alternatives (Scheme 1), Lindsey et al. sought to maximize yields under milder conditions by promoting the establishment of an equilibrium for the cyclization step, then adding a gentle oxidizer (*p*-chloranil or 2,3-dichloro-5,6-dicyano-1,4-benzoquinone (DDQ)) in a second step to obtain irreversibly the aromatized porphyrin [11]. The symmetrical tetra-*meso-*substituted porphyrins are synthesized through a four-fold acid-catalyzed condensation to form a porphyrinogen which is then oxidized (− 6H) to form the conjugated porphyrin product. Lindsey’s reaction takes place at room temperature and utilizes chlorinated solvents under optimized dilution conditions (10^−2^ M reactant concentration). The cyclization step necessitates an oxygen-free environment, in order to allow the equilibrium to be fully established before any oxidation takes place. Tar-like byproducts are avoided under these conditions, and purification is achieved using only a short chromatography column.

Our previous work on mechanochemical porphyrin synthesis has demonstrated that it is possible to synthesize tetraphenylporphyrin (TPP) by grinding benzaldehyde and pyrrole (two liquids) in the presence of an acid catalyst, followed by oxidation with DDQ in minimal amounts of solvent [19]. TPP was produced in a yield similar to that obtained from the Lindsey synthesis. Additionally, several monosubstituted benzaldehydes, as well as two isomers of naphthaldehyde, were shown to undergo successful mechanochemical reaction in reasonable yields.

Mesitaldehyde has been considered a representative “sterically-hindered” reagent, serving as a touchstone for the wider applicability of porphyrin reaction conditions [12]. Porphyrins with bulky groups in the *meso*-positions, especially aromatics with bulky substituents in the position *ortho* to the methine bridge, have been used for biological modelling. The large groups become oriented above and below the plane of the porphyrin ring, forming a protective shroud around the reactive center, much like some protein systems (Figure 1B) [12,20–22]. The corresponding substituted benzaldehydes, however, have not easily yielded results upon reaction with pyrrole under any traditional synthetic method. High-temperature (>170 °C) sealed-bomb reactions in the presence of metal salts will, after 2–3 days, yield 1–6% of *meso*-tetrakis[2,4,6-(trimethyl)phenyl]porphyrin (TMP) [20–21,23–24], and a gas-phase synthesis in the presence of TFA yielded 7% TMP [9]. Under the first-reported conditions of the Lindsey synthesis, mesitaldehyde “failed to give good yields” of TMP [11]. Later studies of modified reaction conditions employing boron trifluoride as the acid catalyst, and small amounts of ethanol as a co-catalyst, were successful in producing TMP with yields around 30% at room temperature [13,25–26]. Optimized conditions still required reactant concentrations of 10^−2^ M. Increasing or decreasing the concentration lowered the yield, as did increasing the temperature. In the present study we set out to test whether our mechanochemical porphyrin synthesis could accommodate a sterically-hindered aldehyde, and produce TMP at room temperature and in the absence of added solvent for the condensation step, using relatively simple reaction conditions. The use of mechanochemistry to bring about a no-solvent-added acid-catalyzed condensation between aldehydes and pyrrole enables the reduction of solvent and elimination of high temperatures from the synthesis of these important compounds, representing a significant reduction in environmental impact.

## Results and Discussion

**Scheme 2:**
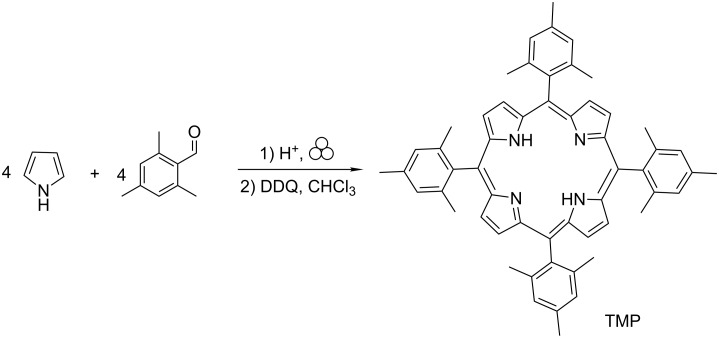
Mechanochemical synthesis of tetramesitylporphyrin.

When mesitaldehyde and pyrrole, both colorless liquids, are combined in the presence of an acid catalyst and ground using a mixer mill for 10 minutes (Scheme 2), a pink powder is formed. As in the case of benzaldehyde, the powder is found to contain no porphyrin, evidenced by the lack of a Soret band at 410–420 nm in the electronic spectrum. Our work with benzaldehyde showed that even when grinding is carried out manually and open to air using a mortar and pestle, no Soret band is observed in the spectrum of the freshly-ground powder [19]. Also in the case of benzaldehyde, it was found that allowing the mechanochemically-generated powder to sit on a benchtop open to air for several weeks brought about the appearance of TPP in small amounts. More immediate oxidation of this power, by dissolving in chloroform and stirring with DDQ for 2 hours, allowed isolation of TPP in 28% yield [19]. Appearance of TPP upon oxidation confirms that mechanochemistry successfully brought about the condensation step. The freshly-ground “pink powder” must contain cyclized products, perhaps including porphyrinogen (colorless) and various other reduced-porphyrin intermediates having one to five more hydrogens than TPP. Many of these intermediates have absorbance in the visible region.

Other no-solvent-added routes to porphyrins, including high-temperature sealed-bomb methods [15–17], gas-phase synthesis [9], and microwave irradiation [10] do not employ a second oxidation step. Presumably for the latter two methods open to air, dioxygen serves as a rapid oxidizer under high-temperature conditions, while the sealed-bomb reaction products are heavily contaminated with chlorins [27–29], byproducts that contain two more hydrogens than the porphyrin, and which easily undergo chemical oxidation to yield porphyrin.

In contrast, since the no-solvent-added mechanochemical synthesis takes place at room temperature, like the Lindsey synthesis, it may be that the equilibrium is reached between starting materials and cyclocondensation products before conditions are introduced to trigger the irreversible oxidation of porphyrinogen to porphyrin. Optimization of conditions could maximize the porphyrinogen yield by adjusting the equilibrium, even though the absence of added solvent means the concentration of reagents is at a maximum, and high concentrations are usually thought to favour linear over cyclic products.

In the present work for the mesitaldehyde reaction, the pink powder obtained upon grinding was immediately combined with DDQ, dissolved in a small amount (ca. 50 mL) of chloroform, and left to stir for one week. This is the same oxidation method as used by Lindsey et al. and is presumed in their case to convert any porphyrinogen or partially-oxidized intermediate present into porphyrin instantaneously [11]. In the case of the mechanochemical synthesis, the results show that a longer oxidation time results reproducibly in a somewhat higher yield (Table 1), although the isolated yield remains around 5%. Identity and purity of TMP was confirmed by UV–vis and ^1^H NMR spectroscopy (see Supporting Information File 1). Although we are not certain of the identity of intermediates or side products from the mechanochemical reaction, it may be possible that via milling they are activated enough so that mechanical stirring during the oxidation with DDQ in solution continues to promote the formation of porphyrinogen. Throughout the oxidation process, the porphyrinogen immediately gets irreversibly oxidized to porphyrin. It is clear that further work is needed to elucidate differences between the mechanochemical and the solvent-based porphyrin syntheses.

**Table 1:** Tetramesitylporphyrin yields.

Milling time (minutes)	Oxidation time^a^	Average isolated yield (%)^b^

10	1 week	1.82
10	1 month	5.10

^a^Stirring in chloroform with DDQ; ^b^Average based on isolated, purified mass from three replicates.

## Conclusion

Mechanochemical milling has successfully led to cyclocondensation of pyrrole with a sterically-hindered aldehyde. After oxidation to give a porphyrin with bulky substituents, isolated yields are comparable to those obtained from high-temperature syntheses (though still lower than the 30% obtainable with added solvent at room temperature). The mechanochemical synthesis is carried out at room temperature with no added solvent during the condensation step, simplifying and reducing the environmental impact for the synthesis of this important class of molecules. Notably, James et al. have reported the simple and clean mechanochemical metalation of porphyrins, extending these advantages even further [30]. Further studies aimed at understanding the mechanism of the mechanochemical porphyrin synthesis and its differences and similarities compared to the solvent-based methods will be important advances for the field of mechanochemistry.

## Experimental

### Materials and methods

All chemicals used were purchased from Sigma-Aldrich and used without further purification. Mesitaldehyde (98%), *p*-toluenesulfonic acid monohydrate (*p*-TSA, 98.5%), chloroform (99.8%), alumina (99.9%), triethylamine (99.5%), silica gel (technical grade, pore size 60 Å, 200–425 mesh particle size), ethyl acetate (anhydrous, 99.8%), hexane (mixture of isomers, 98.5%), deuterated chloroform (99.8 atom % D) were used. 2,3-Dichloro-5,6-dicyano-1,4-benzoquinone (DDQ, 97.0%) was purchased from TCI America and used without further purification. Pyrrole (98%) was purchased from Sigma-Aldrich and purified by distillation once per week after discoloration appeared. The electronic spectra were recorded on a Perkin Elmer Lambda 850 UV–vis spectrophotometer, measured from 200–800 nm at 1 nm intervals. The samples were placed in quartz cuvettes with a 1 cm path length. ^1^H NMR spectra were recorded on a Varian 300 MHz nuclear magnetic resonance spectrometer.

### Mechanochemical synthesis

***meso*-Tetrakis[2,4,6-(trimethyl)phenyl]porphyrin.** Equimolar amounts of pyrrole (0.259 g, 3.75 mmol) and 2,4,6-trimethylbenzaldehyde (0.556 g, 3.75 mmol) were placed in a 10 mL stainless steel grinding jar along with acid catalyst *p*-toluenesulfonic acid (0.026 g, 0.151 mmol, 4%) and two stainless steel balls of 5 mm diameter (0.52 g/ball; 0.81 mass-to-balls ratio). The mixture was ground using the Retsch Mixer Mill MM200 for 10 minutes at a frequency of 25 Hz, resulting in formation of a dark pink-colored powder. The powder was then dissolved in 50 mL chloroform and 3 molar equivalents of DDQ (2.554 g, 11.20 mmol) were added. The mixture was stirred to undergo oxidation for a period of one week. The mixture was then passed through a 1 cm alumina plug and 3–5 drops of triethylamine were added to the filtrate. The filtrate was passed through a silica gel column using 1:3 ethyl acetate/hexanes as the mobile phase. Fractions containing porphyrin, as determined by thin-layer chromatography and UV–vis spectroscopy were combined and the solvent was removed using a rotary evaporator. *meso*-Tetrakis[2,4,6-(trimethyl)phenyl]porphyrin was isolated as a purple-red crystalline powder in 1.8% average yield, which could be increased to 5.1% if oxidation time was lengthened to one month. ^1^H NMR (300 MHz, CDCl_3_) δ 8.62 (s, 8H), 6.91 (s, 8H), 2.62 (s, 12H), 1.85 (s, 24H), −2.52 (s, 2H); ^13^C NMR (300 MHz, CDCl_3_) δ 138.4, 137.2, 136.6, 126.7, 116.5, 37.1, 30.9; UV–vis (CHCl_3_) λ = 414 (Soret band), 513, 543, 590, 648 (Q-bands).

## Supporting Information

File 1 Spectroscopic characterization of *meso*-tetrakis[2,4,6-(trimethyl)phenyl]porphyrin (TMP).
