# Supplementary material for: Extending mechanochemical porphyrin synthesis to bulkier aromatics: tetramesitylporphyrin
Source: Beilstein J Org Chem. 2019 May 22;15:1149–53. doi: 10.3762/bjoc.15.111 (PMC6541328; doi:10.3762/bjoc.15.111)
Supplement: File 1 — Spectroscopic characterization of meso-tetrakis[2,4,6-(trimethyl)phenyl]porphyrin (TMP). [file Beilstein_J_Org_Chem-15-1149-s001.pdf]

BEILSTEIN JOURNAL OF ORGANIC CHEMISTRY

Supporting Information

for

Extending mechanochemical porphyrin synthesis to bulkier aromatics: tetramesitylporphyrin

Qiwen Su and Tamara D. Hamilton

Beilstein J. Org. Chem. **2019,** *15,* 1149–1153. doi:10.3762/bjoc.15.111

Spectroscopic characterization of *meso*-tetrakis[2,4,6-(trimethyl)phenyl]porphyrin (TMP)

License and Terms: This is a supporting information file under the terms of the Creative Commons Attribution License (http://creativecommons.org/licenses/by/4.0). Please note that the reuse, redistribution and reproduction in particular requires that the authors and source are credited.
The license is subject to the *Beilstein Journal of Organic Chemistry* terms and conditions: (https://www.beilstein-journals.org/bjoc)

Table of Contents

A. UV–vis spectrum of TMP……………………………..…....……S2

B. ^{1}H NMR spectrum of TMP……………………………………....S3

C. ^{13}C NMR spectrum of TMP………………………………..…..S4

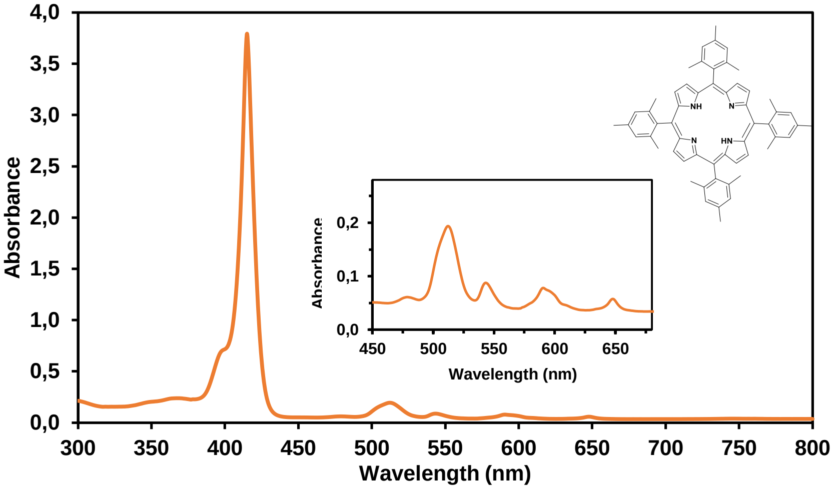

Figure S1: UV–vis spectrum of *meso*-tetrakis[2,4,6-(trimethyl)phenyl]porphyrin. λ = 414 (Soret band); λ = 513, 543, 590, 648 (Q-bands).

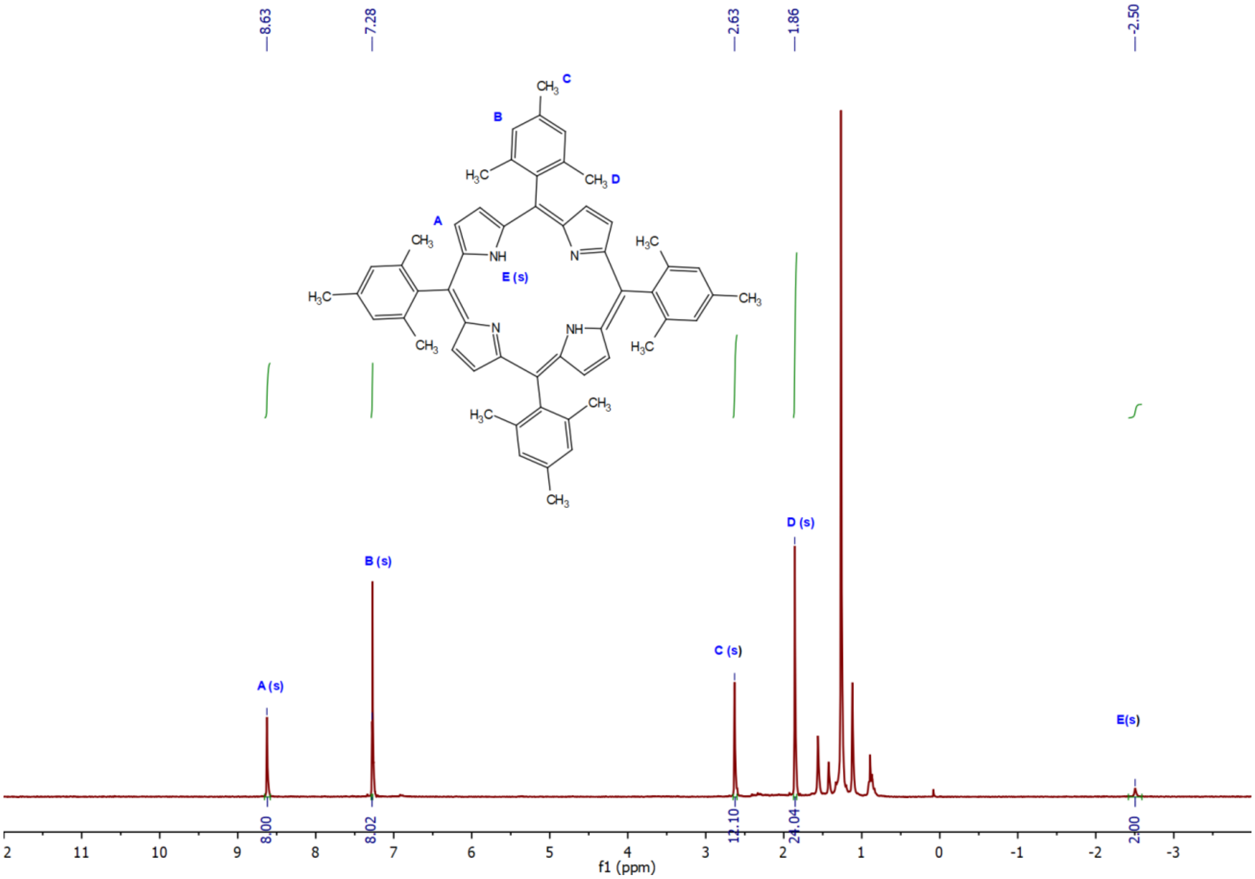

Figure S2: ^{1}H NMR spectrum of *meso*-tetrakis[2,4,6-(trimethyl)phenyl]porphyrin. (300 MHz, $CDCl_3$): δ = 8.63 (s, 8H), 7.28 (s, 8H), 2.63 (s, 12H), 1.86 (s, 24H), -2.50 (s, 2H).

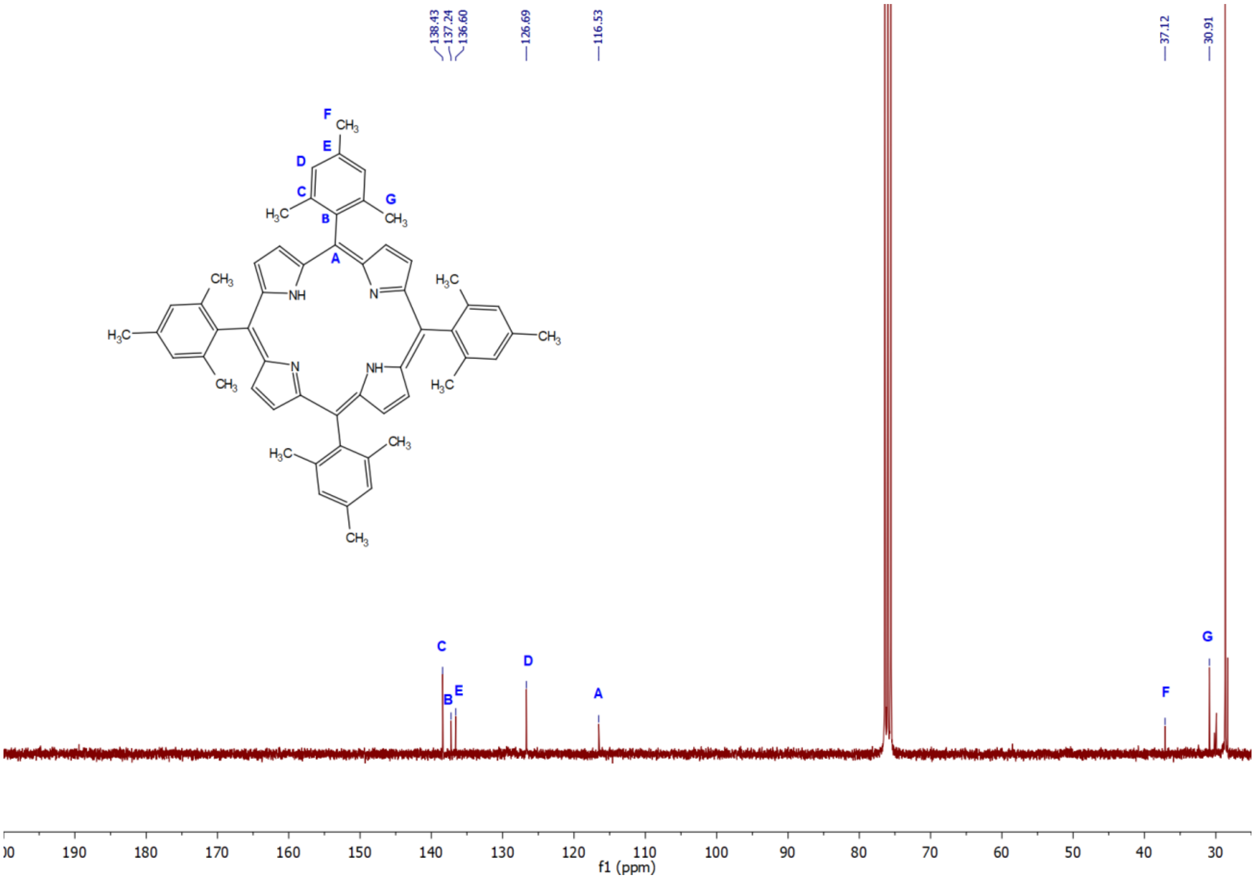

Figure S3: ^{13}C NMR spectrum of *meso*-tetrakis[2,4,6-(trimethyl)phenyl]porphyrin (300 MHz, CDCl3): δ= 138.4, 137.2, 136.6, 126.7, 116.5, 37.1, 30.9.

Note: In ^{13}C NMR spectra of free base porphyrins, the α and β carbons on the pyrrole rings are often indistinguishable from baseline noise or too broad to be observable.[1]

Reference:

1) Mohajer, D.; Zakavi, S.; Rayati, S.; Zahedi, M.; Safari, N.; Khavasi, H. R.; Shahbazian, S. *New J. Chem.* **2004**, *28*, 1600-1607.
